# Epilepsy is related to theta band brain connectivity and network topology in brain tumor patients

**DOI:** 10.1186/1471-2202-11-103

**Published:** 2010-08-23

**Authors:** Linda Douw, Edwin van Dellen, Marjolein de Groot, Jan J Heimans, Martin Klein, Cornelis J Stam, Jaap C Reijneveld

**Affiliations:** 1 Department of Neurology, VU University Medical Center, Amsterdam, The Netherlands; 2 Department of Medical Psychology, VU University Medical Center, Amsterdam, The Netherlands; 3 Department of Clinical Neurophysiology, VU University Medical Center, Amsterdam, The Netherlands

## Abstract

**Background:**

Both epilepsy patients and brain tumor patients show altered functional connectivity and less optimal brain network topology when compared to healthy controls, particularly in the theta band. Furthermore, the duration and characteristics of epilepsy may also influence functional interactions in brain networks. However, the specific features of connectivity and networks in tumor-related epilepsy have not been investigated yet. We hypothesize that epilepsy characteristics are related to (theta band) connectivity and network architecture in operated glioma patients suffering from epileptic seizures. Included patients participated in a clinical study investigating the effect of levetiracetam monotherapy on seizure frequency in glioma patients, and were assessed at two time points: directly after neurosurgery (t1), and six months later (t2). At these time points, magnetoencephalography (MEG) was recorded and information regarding clinical status and epilepsy history was collected. Functional connectivity was calculated in six frequency bands, as were a number of network measures such as normalized clustering coefficient and path length.

**Results:**

At the two time points, MEG registrations were performed in respectively 17 and 12 patients. No changes in connectivity or network topology occurred over time. Increased theta band connectivity at t1 and t2 was related to a higher total number of seizures. Furthermore, higher number of seizures was related to a less optimal, more random brain network topology. Other factors were not significantly related to functional connectivity or network topology.

**Conclusions:**

These results indicate that (pathologically) increased theta band connectivity is related to a higher number of epileptic seizures in brain tumor patients, suggesting that theta band connectivity changes are a hallmark of tumor-related epilepsy. Furthermore, a more random brain network topology is related to greater vulnerability to seizures. Thus, functional connectivity and brain network architecture may prove to be important parameters of tumor-related epilepsy.

## Background

Gliomas are primary brain tumors arising from the supporting tissue of the brain. The presenting symptoms in glioma patients include epileptic seizures in 20-45% of patients [1]. Another 15-30% of patients develop seizures during their disease [2]. Multiple factors may contribute to epileptogenesis (for a review, see [3]), but all possible factors that have been studied up till now do not suffice when trying to clarify the course of tumor-related epilepsy. Possibly, a better understanding of tumor-related epilepsy could be achieved using a relatively new concept in neuroscience: 'functional connectivity' refers to the statistical interdependencies between neurophysiological time series such as EEG, MEG, or fMRI-BOLD signals [4,5]. Functional connectivity is thought to reflect communication between different brain areas, thus having a major impact on brain functioning [6,7]. Furthermore, the brain can be seen as a complex integrated network, in which focal changes influence the integrity and status of the brain as a whole. An 'optimal' brain network probably includes concepts that are pivotal in many types of complex networks, such as localized segregation combined with overall integration [6,8,9]. Watts and Strogatz proposed a theoretical framework for such a network, the so-called 'small-world' network [10]. The small-world network is an intermediate type of network in between the 'random' network, in which all connections between nodes are randomly drawn, and the 'regular' network, in which connections between nodes are present in an ordered fashion and all nodes have the same number of connections. Several studies have shown that both structural and functional brain networks in healthy humans and animals can be characterized by the small-world principle [10-13].

Previous studies using magnetoencephalography (MEG) recordings show that functional connectivity and network topology are significantly altered in brain tumor patients when compared to healthy participants: low frequency connectivity (in particularly the theta band) is pathologically increased, and the normal small-world configuration is disturbed [14-17]. These differences are not only limited to the area around the tumor, but involve brain-wide networks and are related to cognitive deficits. Altered connectivity and network topology have also been reported in epilepsy patients, even in the inter-ictal period. Increased theta, but also delta, frequency EEG connectivity in between seizures was found in two studies investigating epilepsy patients [18,19]. Furthermore, therapy-resistant epilepsy patients have been reported to have a more regular network topology than healthy controls as measured with EEG [19].

Changes over time in connectivity and network patterns in neuro-oncological or epilepsy patients have only been sparsely reported. In a previous study, we investigated a patient group with varying types of primary brain tumors with MEG before and after tumor resection [20]. After tumor resection, functional connectivity in the theta band significantly decreased in these patients, suggesting 'normalization' of the previously reported pathologically increased connectivity. Moreover, this decrease was related to better postsurgical outcome in terms of seizure-freedom. Studies using both fMRI and acute corticography (ACoG) recordings in mTLE patients suggest that prolonged therapy-resistant epilepsy is related to decreases in broadband functional connectivity [21,22]. Furthermore, the small-world architecture is more disrupted in the temporal cortical networks of TLE patients with longer epilepsy history, as measured with ACoG [21].

Previous studies suggest that both epilepsy and brain tumors are related to changes in connectivity, which may be most prominent in the theta band. Furthermore, brain networks of both epilepsy patients and brain tumor patients display a loss of small-world features when compared to healthy controls. In the current study, we first aimed to investigate the relation between functional connectivity, network topology, and tumor-related epilepsy in a group of glioma patients. We hypothesized increased connectivity and less optimal network topology in the theta band to be related to epilepsy characteristics. Secondly, we studied the longitudinal changes in connectivity and network architecture during the first six months after neurosurgery in relation to changes in epilepsy features. The patients participated in a clinical study investigating levetiracetam monotherapy (an anti-epileptic drug (AED)), which implies that patients were homogeneous with respect to AED use.

## Results

### Patients

A total of 24 patients were included, but 7 patients had to be excluded due to MEG artifacts at t1. Further analyses were performed using the remaining 17 patients. All patients had undergone surgical intervention and histopathological diagnoses were obtained (see table 1). Patients' mean age was 44 years (SD = 12 years) and four patients were females. The majority of patients was diagnosed with glioblastoma multiforme (GBM, nine patients). The tumor was localized in the right hemisphere in 12 patients. These tumors were all localized in the frontal or temporal lobe. Five patients had left-sided tumors, which were localized in the frontal lobe in most patients. Most patients (11 of 17) had at most one seizure per month in the last month before t1 (i.e. seizure frequency in table 1), while three patients had more than one seizure per day. Seizures were likely to be due to the tumor in all patients. At t2, 12 patients underwent a second MEG recording: two patients were excluded because they switched to another AED, while one patient had died due to disease progression. In two patients, no artifact-free MEG epochs were available. Of these 12 patients, 10 patients did not have seizures anymore, while 2 patients still had occasional seizures. At t3, only 6 patients were tested. Due to the small number of patients available at t3, no further analysis was performed on these measurements. Patient characteristics at t2 are displayed in table 2.

**Table 1:** Patient characteristics at t1

	Age	Gender	Seizure type	Time since 1st seizure (months)	Seizure frequency (per month)	Total number of seizures	Type of surgery	Histopathological diagnosis	Tumor localization	CT	RT	DEX	KPS	Barthel
1	57	M	PS	65	4-30	10	R	OIII	RF	N	Y	N	90	20
2	43	M	GS	19	> 30	120	R	AII	RF	Y	N	N	70	19
3	46	F	GS	163	0	11	R	OII	LF	Y	N	N	90	20
4	49	M	GS	30	> 30	20	R	GBM	LF	Y	Y	N	80	20
5	68	M	PS	61	> 30	2	R	GBM	RT	Y	N	Y	100	20
6	36	M	GS	5	0	1	R	AIII	RF	N	Y	N	90	20
7	30	M	GS	21	1	2	R	AII	RF	N	N	N	100	20
8	50	M	GS	11	1	1	B	AIII	LPO	Y	Y	Y	90	20
9	25	F	GS	15	4-30	8	B	GBM	RT	Y	Y	Y	90	20
10	61	M	GS	32	0	1	R	GBM	RF	Y	Y	Y	100	20
11	45	M	GS	117	1	1	R	OIII	RT	N	Y	N	90	20
12	37	F	GS	33	1	2	R	GBM	RFT	Y	Y	N	90	20
13	47	M	GS	38	1	3	R	AIII	LF	N	Y	N	70	19
14	29	M	GS	22	1-4	3	R	GBM	RF	Y	Y	N	90	20
15	49	F	PS	9	1	2	R	GBM	LF	Y	Y	Y	80	16
16	48	M	GS	10	0	1	R	GBM	RT	Y	Y	N	100	20
17	25	M	GS	16	1	3	R	GBM	RT	Y	Y	N	100	20

*Note*. CT = chemotherapy, RT = radiotherapy, DEX = dexamethasone, KPS = Karnofsky performance status, M = male, F = female, PS = partial seizures, GS = generalized seizures, R = (sub)total resection, B = stereotactic biopsy, OII or III = oligodendroglioma WHO grade II or III, AII or III = astrocytoma WHO grade II or III, GBM = glioblastoma multiforme, RF = right frontal, LF = left frontal, RT = right temporal, LPO = left parieto-occipital, RFT = right fronto-temporal, N = no, Y = yes.

**Table 2:** Patient characteristics at t2

	Seizure frequency (per month)	Total number of seizures	CT	RT	DEX	KPS	Barthel
1	0	10	N	N	N	100	19
3	0	11	N	N	N	90	20
4	1	22	Y	N	Y	80	20
6	0	1	N	N	N	90	20
7	0	2	N	N	N	100	20
8	1	3	Y	N	Y	100	20
9	0	8	N	N	Y	90	20
10	0	1	Y	N	N	90	20
11	0	1	N	N	N	90	20
12	0	2	Y	N	N	80	20
15	0	2	N	N	N	80	18
16	0	1	Y	N	N	90	20

*Note*. CT = chemotherapy, RT = radiotherapy, DEX = dexamethasone, KPS = Karnofsky performance status, N = no, Y = yes.

### Power analysis

Relative power in each frequency band was calculated. No significant changes in relative power occurred between t1 and t2. There were also no correlations between relative power in each frequency band and clinical characteristics, such as seizure frequency and total number of seizures.

### Functional connectivity

**Figure 1:**
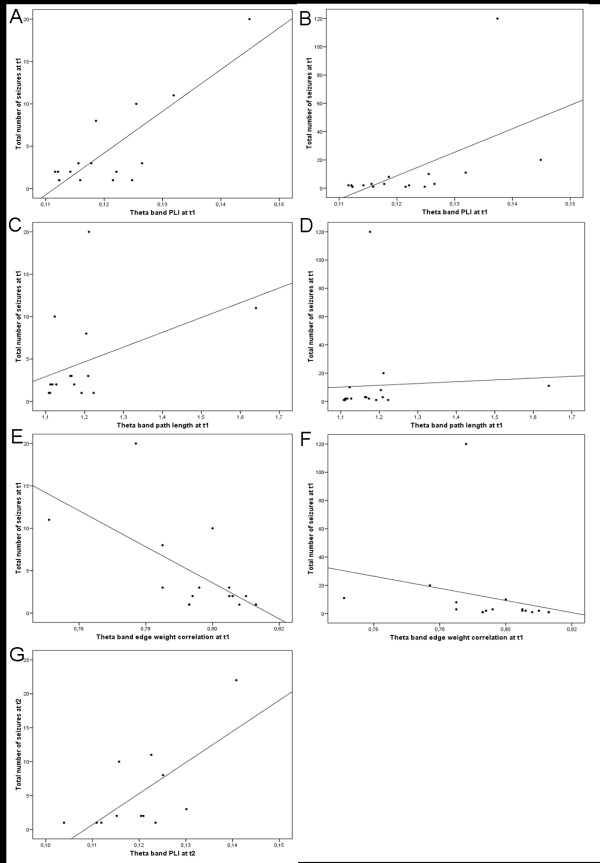
**Scatterplots of correlations between functional connectivity, network characteristics, and total number of seizures at both t1 and t2**. *Note*. Left column: scatterplots excluding outlying patients (patient 2 in table 1), right column: scatterplots including this patient. (A) correlation between theta band phase lag index (PLI) at t1 without outlying patient, (B) correlation between theta band PLI at t1 with outlying patient, (C) correlation between theta band path length and total number of seizures at t1 without outlying patient, (D) correlation between theta band path length and total number of seizures at t1 with outlying patient, (E) correlation between theta band edge weight correlation and total number of seizures at t1 without outlying patient, (F) correlation between theta band edge weight correlation and total number of seizures at t1 with outlying patient, and (G) correlation between theta band PLI and total number of seizures at t2.

**Figure 2:**
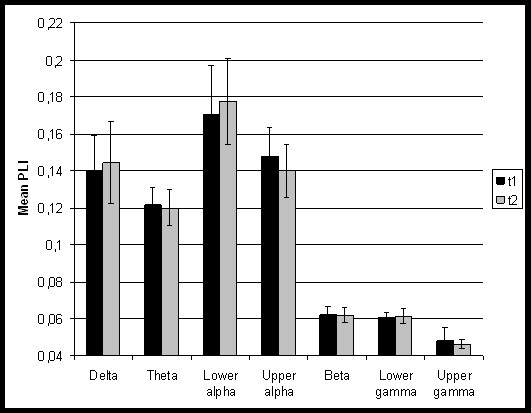
**Mean PLI at both time points**. *Note*. PLI = phase lag index.

Significant correlations were found between functional connectivity and the total number of seizures, which refers to the total number of seizures that patients had experienced from the first to the last seizure before MEG measurement at the first time point. At t1, higher total number of seizures was significantly associated with higher theta band PLI (Kendall's Tau = .501, p = .008; see figure 1A). This correlation remained significant when excluding an outlier patient, who had experienced a total of 120 seizures (this patient was excluded from figure 1). At t2, the same association was found, although significance was lost after correcting for the number of comparisons (Kendall's Tau = 0.538, p = .020; see figure 1D). Seizure frequency significantly dropped between t1 and t2 from on average 11 seizures at t1 to less than one seizure between t1 and t2 (Wilcoxon signed rank test, Z = -2.81, p = .003). There were no significant changes in functional connectivity between the three time points (see figure 2).

In order to explore which type of theta band connectivity was related to the total number of seizures at t1, we summarized connectivity into three values: (1) short-distance PLI, (2) long-distance intrahemispheric PLI, and (3) long-distance interhemispheric PLI (see [20]). There were highly significant correlations between the total number of seizures at t1 with both short-distance theta band PLI (Kendall's Tau = .531, p = .005) as well as with long-distance intrahemispheric theta band PLI (Kendall's Tau = .563, p = .003). Theta band functional connectivity in the temporal lobe seemed to have the strongest relation to the number of seizures: left temporo-occipital PLI (Kendall's Tau = .563, p = .003), right fronto-temporal PLI (Kendall's Tau = .610, p = .001), right temporo-occipital PLI (Kendall's Tau = .531, p = .005), and left temporal PLI (Kendall's Tau = .515, p = .006).

Several possible confounders regarding the reported results were explored in post-hoc analyses. There were no significant associations between functional connectivity and patients' age, the total daily dose of levetiracetam, anti-tumor treatment (chemotherapy, radiotherapy, or dexamethasone), performance status, and the duration of epilepsy at t1. Furthermore, there were no differences in functional connectivity between men and women (gender differences have been reported previously with respect to anatomical and functional connectivity [23,24]), patients with disease recurrence versus newly diagnosed patients, patients suffering from partial or generalized seizures, patients with low-grade versus high-grade tumors, left-sided and right-sided tumors, and type of resection (biopsy or (sub)total resection), although only two patients underwent biopsy.

### Graph analysis

**Figure 3:**
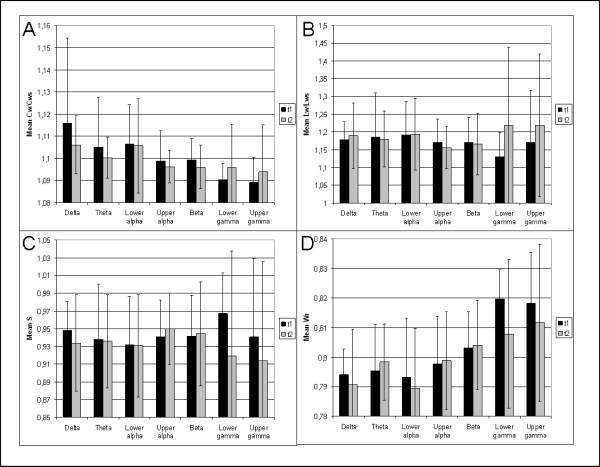
**Mean network characteristics at both time points**. *Note*. C_w_/C_ws _= weighted normalized clustering coefficient, L_w_/L_ws _= weighted normalized clustering coefficient, S = small-world index, W_r _= edge weight correlation.

Increasing theta band edge weight correlation (i.e. lower value of W_r_) was significantly related to higher total number of seizures at t1 (Kendall's Tau = -.507, p = .008; see figure 1C). Higher total number of seizures at t1 was related to higher theta band path length, although this correlation was not significant after correcting for multiple comparisons (Kendall's Tau = .404, p = .031; see figure 1B). There were no significant changes in network features between the two time points (see figure 3).

Again, confounders were explored. There was no correlation between network features and duration of epilepsy, tumor treatment (radiotherapy, chemotherapy, dexamethasone), performance status, and daily dose of levetiracetam. There were no differences in network features between male and female patients, patients with recurrent disease or newly diagnosed brain tumors, patients who had undergone resection versus biopsy, patients with tumors in the left and right hemisphere, and patients with partial seizures and patients with generalized seizures. However, higher age was related to lower edge weight correlation (Kendall's Tau = .502, p = .006).

## Discussion

Significant associations exist between epilepsy characteristics and both functional connectivity and network topology in brain tumor patients directly after neurosurgical intervention: increased theta band phase lag index (PLI) is related to a greater total number of seizures. This association mostly concerns theta band connectivity within the temporal lobe, and between the temporal and other lobes. There is also a correlation between higher total number of seizures and higher theta band edge weight correlation (W_r_). No significant changes in functional connectivity or network variables were observed over time. Furthermore, the total number of seizures from the first seizure to both time points seemed to be the only factor related to differences in connectivity and network variables (except age), while other variables such as treatment characteristics, type of tumor, and lateralization of the tumor were not significantly related to connectivity and network topology.

Our findings concerning the association between theta band functional connectivity and number of epileptic seizures corroborate several lines of previous research and confirm our initial hypothesis. Earlier studies in brain tumor patients have shown that these patients have increased connectivity in lower frequency bands, and particularly in the theta band [14,15]. Furthermore, theta band functional connectivity significantly decreased after tumor resection in a group of brain tumor patients, suggesting 'normalization' of the previously reported pathologically increased connectivity [20]. In epilepsy, increased theta band connectivity has also been reported in the inter-ictal EEG [18]. Increased theta band functional connectivity seems to be a hallmark of epilepsy, tumor-related epilepsy, and/or brain tumors, and the current results stimulate further investigations into this hypothesis.

Brain tumor patients have been found to have disrupted small-world brain networks when compared to healthy controls [16,17]. In the current study, there was a significant relation between higher number of seizures and higher edge weight correlation in the theta band. Although high edge weight correlation has been thought to be beneficial to functional status of the network, because of increased transport of information [25], correlations that are too high may increase vulnerability to seizures due to an abnormally high synchronisability of the brain network. Using a model of rat hippocampus, it has been shown that adding highly connected hubs increases network vulnerability to seizures [26]. Furthermore, single-cell recordings in rats also show that hubs are highly influential throughout the network, and may or may not promote synchronisability [27]. At seizure onset, epilepsy patients also show increases of average interconnectedness of the network [28-30]. Thus, it seems as though there is a critical threshold of connectivity and synchronisability. There was also a trend towards a correlation between higher total number of seizures and longer theta band path length. This increased path length in epilepsy patients has been found with EEG [19].

The associations between connectivity and seizures in this study were mainly due to connectivity with and within the temporal lobe. This result could be due to the relatively high number of patients with temporal tumors (6 of 17), but may also be related to the origins of theta band oscillations and its relation with epilepsy. The theta band contains an oscillatory pattern that has for long been thought to emanate from the hippocampal structures, after which it spreads to the outer layers of the brain. However, later studies have shown that other regions of the brain may also generate theta oscillations in certain cognitive states [31]. Power, amplitude, and synchronization of the theta band has mainly been linked to cognitive functioning, particularly processes involved in learning and memory [32-34]. Microscopically, theta band oscillations may be regulated by GABAergic interneurons [35,36]. Moreover, blockade of GABA receptors in induced epilepsy alters patterns of theta activity [37], and hubs consisting of GABAergic interneurons may determine network synchronization [27]. Also, neuronal changes associated with temporal lobe epilepsy can disrupt hippocampal theta function [38]. These studies point towards a possible link between epilepsy and the theta band. In human neurophysiological data, the association between the theta band and epilepsy has increasingly been studied over the past two decades. Pathological thalamo-cortical theta oscillations have been described in absence seizures [39], and increased theta band absolute spectral power is related to absence epilepsy [40] and generalized epilepsies [41]. Another study reports increased theta band power to be related to the severity of epilepsy [42]. Moreover, interhemispheric theta band coherence proved to be a selective feature of patients with generalized epilepsy [39].

Functional connectivity and network topology did not change significantly over the two time points in this study, although seizure frequency did change over time. The limited sample size in this study may have impacted significance, but the lack of change may also be due to the possibility of a stable plateau phase in connectivity and network topology. Possibly, resection of the tumor induces a great change in functional connectivity [20], after which this stable phase is reached at t1, approximately 6 weeks after neurosurgical intervention. Future studies should aim at elucidating this possibility. A number of explored variables also did not have a significant impact on connectivity and network features, although this intuitively may have been expected. This could be due to the small sample size and heterogeneous group, but the impact of radiotherapy, chemotherapy, and dexamethasone on the background EEG is largely unknown. A study investigating epilepsy patients receiving add-on levetiracetam treatment reports no changes in background EEG, although they did not look at connectivity [43]. Furthermore, there was no significant influence of the type (i.e. WHO grade) and lateralization (left or right hemisphere) of the tumor on functional connectivity or network characteristics, corroborating previous research that has also pointed out that the location of the tumor does not determine the pattern of connectivity changes in the brain, although patients with right-sided tumors were generally better off in terms of alterations in connectivity [15]. Another possible explanation for the lack of change may also be the high heritability of both functional connectivity and network topology [44,45]: the effect of factors such as tumor treatment may not be strong enough to overcome the constancy of the genetically determined network architecture.

This study has some limitations, one of which is the small sample size, limiting statistical power. Although patients were homogeneous with respect to brain tumor type (glioma) and AED, variation was present regarding a number of variables. These variables were not significantly related to functional connectivity or network features, but future studies should aim to investigate more homogeneous subgroups of brain tumor patients to elucidate more specific effects. Furthermore, all MEG experiments have the limitation of the inverse problem, common sources and volume conduction. However, the PLI is very strict in this respect and disregards all zero-lagged correlations, which means that our results are not the result of spurious correlations because of common sources or volume conduction.

## Conclusions

Our results suggest that theta band connectivity and network topology may be important for tumor-related epileptic seizures. These findings bring up thoughts about possible mechanisms of epileptogenesis in brain tumor patients. Functional connectivity and network characteristics in the theta band seem most important in tumor-related epilepsy. Future research should focus on elucidating this correlation between epilepsy and the theta band.

## Methods

### Patients

This study was performed on patients who were included in a study primarily investigating the effect of levetiracetam monotherapy in brain tumor patients. Between April 1^st ^2007 and June 1^st ^2009, patients were recruited from two tertiary referral centers for brain tumor patients in The Netherlands (VU University Medical Center and Academic Medical Center, Amsterdam). Inclusion criteria were: (1) a diagnosis of novel or recurrent glioma confirmed by pathological diagnosis, (2) age ≥ 18 years, and (3) generalized or partial seizures with or without secondary generalization. All patients had undergone surgery and were treated with levetiracetam monotherapy at the time of inclusion. Exclusion criteria were: (1) lack of a basic proficiency of the Dutch language, or (2) the inability to communicate adequately. The first MEG recordings took place within six weeks after neurosurgery (t1). Follow-up took place after six months (t2) and one year (t3). Data regarding medical status, physical examination, and laboratory investigations were collected at these time points, as well as Karnofsky performance status [46] and Barthel index [47]. Patients were excluded during follow-up if their treating neurologist decided to discontinue levetiracetam monotherapy or if another antiepileptic drug (AED) was added to their regime. All patients gave written informed consent before participating, and study approval was obtained from both centers' ethics committees.

### Magnetoencephalography (MEG)

Magnetic fields were recorded while subjects were seated inside a magnetically shielded room (Vacuumschmelze GmbH, Hanau, Germany) using a 151-channel whole-head MEG system (CTF Systems Inc., Port Coquitlam, BC, Canada). A third-order software gradient [48] was used with a recording pass band of 0.25-125 Hz. Fields were measured during a no-task eyes-closed condition, with a sample frequency of 625 Hz. At the beginning and end of each recording, the head position relative to the coordinate system of the helmet was recorded by leading small alternating currents through three head position coils attached to the left and right pre-auricular points and the nasion on the patient's head. Head position changes up to approximately 1.5 cm during a recording condition were accepted. During the recording, patients were instructed to close their eyes to reduce artifact signals due to eye movements. MEG channels that were defective or contained artifacts in at least one patient were excluded in the entire group, leaving 140 of the 151 MEG channels to be included.

### Power analysis

Relative power was calculated by means of Fast Fourier Transformations in six frequency bands (respectively delta (0.5-4 Hz), theta (4-8 Hz), lower alpha (8-10 Hz), upper alpha (10-13 Hz), beta (13-30 Hz), and gamma (30-45 Hz) [49].

### Functional connectivity

Functional connectivity was assessed with the phase lag index (PLI; [50]). The PLI calculates synchronization between time series by reflecting the consistency with which one signal is phase leading or lagging with respect to another signal. The PLI exploits the asymmetry of the distribution of instantaneous phase differences between two signals. It assumes that the presence of a consistent, nonzero phase lag between two time series cannot be explained by volume conduction alone. Thus, finding true interactions instead of volume conduction effects is more likely when using this method. The PLI ranges between 0 and 1, and a PLI of more than 0 indicates phase locking to a certain extent, whereas a PLI of 0 indicates no coupling or coupling with a phase difference centered around 0 ± π radians. An index of the asymmetry of the phase difference distribution can be obtained from a time series of phase differences ΔΦ (t_k_), k = 1... N in the following way:

(1) $$PLI=|\langle sign[\Delta \phi (t_{k})]\rangle |$$

See [50] for a complete description of PLI calculation.

Five artifact free epochs of 4096 samples (6.552 seconds) during resting-state with eyes closed were carefully selected by visual analysis from each patient at each time point [EvD]. PLIs between each pair of MEG sensors were computed after filtering the MEG signals in six frequency bands Computation of the PLI was done offline with DIGEEGXP software, developed at our department [CJS]. PLI for all sensor pairs was averaged over each set of the selected five epochs, after which graph analysis took place.

### Graph analysis

A graph is a topographical representation of a network, constructed by nodes ('vertices') and links ('edges') between them. Graphs can be unweighted (binary) or weighted; in the former case, a threshold is applied for every edge. When the connectivity value is higher than the threshold, the edge is present and gets a value of 1; if not, the edge is not and thus is given a value 0. In this study, we used undirected weighted graphs, in which the weight of every edge was the PLI value of the link between the two nodes it connects.

A wide range of network measures can be calculated after representing MEG data as a graph (see [7,51] for recent reviews). In this study, the most commonly used measures are employed. The first measure is the weighted clustering coefficient C, which refers to the likelihood that neighbors of a vertex will also be connected. The clustering coefficient characterizes the tendency of nodes to form local clusters and is thus a measure of local segregation of the graph. The second measure is the average weighted path length L, signifying the average highest connectivity of edges connecting any two vertices, and is a measure for global integration of the network. The combination of high local clustering and a short average path length seems to be the optimal configuration for efficient communication in a network [10]. A 'small-world' network, which is thought to be a feasible model for human brain networks, has such a configuration (see figure 1). For a more detailed description of calculation of the weighted clustering coefficient C_w _and weighted average shortest path length L_w _in this study see [52].

The (weighted) clustering index of vertex i with edge w with other vertices is defined as:

(2) $$C_{i}=\frac{\sum_{k\neq i}\sum_{\begin{array}{l} l\neq i\\ l\neq k \end{array}}w_{ik}w_{il}w_{kl}}{\sum_{k\neq i}\sum_{\begin{array}{l} l\neq i\\ l\neq k \end{array}}w_{ik}w_{il}}$$

Subsequently, all clustering coefficients of the network are averaged to obtain one C for the entire brain. The weighted path length is calculated as follows:

(3) $$L_{w}=\frac{1}{(1/N(N-1))\sum_{i=1}^{N}\sum_{j\neq i}^{N}\left(1/L_{ij}\right)}$$

We normalized all network characteristics to those of 1000 surrogate random networks of the same size, resulting in the measures C_w_/C_ws_, L_w_/L_ws_. The surrogate networks were obtained from the original networks by randomly reshuffling the edge weights, hereby preserving the symmetry of the matrix.

A second-order graph property has been proposed [53]: the ratio between C_w_/C_ws _and L_w_/L_ws_, which is an index of 'small-worldness'. Graphs with a small-world index > 1 are considered small-world, since C_w_/C_ws _> 1 and L_w_/L_ws _~ 1 apply in small-world networks.

Finally, we calculated the 'edge weight correlation'. This is a measure for the correlation between weights of neighboring edges, i.e. edges that connect to the same vertex [25]. The edge weight correlation is calculated as the range between the highest and lowest weight of all edges per vertex:

(4) $$r_{i}=\frac{W_{\max}(i)-W_{\min}(i)}{W_{\max}(i)+W_{\min}(i)}$$

W_max _accounts for the maximum weight and W_min _for the minimum weight of the edges of node *i*. This range is then compared to that of the random equivalent of the network, in which the edges are randomly redistributed over the vertices while their weights are kept unchanged (as described above for other network characteristics). When the resulting value W_r _lies between 0 and 1, a positive weight-weight correlation exists (because the range of neighboring weight values is smaller than in a random network), whereas the weights are anti-correlated when W_r _> 1. It has been shown that a positive weight correlation (thus: W_r _< 1) dramatically increases transport over the network, and edge weight correlation and thus transport increase further as W_r _approaches 0 [25].

### Statistical analysis

All statistical analyses were performed using SPSS 15.0 for Windows. Functional connectivity and network variables usually do not follow a normal distribution, warranting non-parametric testing. Correlations between connectivity, network features, and seizure-related variables were tested using Kendall's Tau. Differences in connectivity and network topology according to seizure-related variables were tested using Mann-Whitney U-tests. Changes in seizure characteristics, functional connectivity, and network features between the three time points were tested with Wilcoxon signed rank tests. When applicable, we corrected for the number of comparisons with the false discovery rate (FDR [54]).

## Authors' contributions

LD, ED, MG, JJH, MK, CJS, and JCR designed the study. LD, ED, and MG acquired and analyzed the data. LD, ED, MG, JJH, MK, CJS, and JCR interpreted data, and wrote or revised the manuscript.

## References

[B1] Wen PY Schiff D Kesari S Drappatz J Gigas DC Doherty L Medical management of patients with brain tumors J Neurooncol 2006 80 3 313 332 10.1007/s11060-006-9193-2 16807780

[B2] Krouwer HG Pallagi JL Graves NM Management of seizures in brain tumor patients at the end of life J Palliat Med 2000 3 4 465 475 10.1089/jpm.2000.3.4.465 15859699

[B3] Beaumont A Whittle IR The pathogenesis of tumour associated epilepsy Acta Neurochir (Wien) 2000 142 1 1 15 10.1007/s007010050001 10664370

[B4] Aertsen AM Gerstein GL Habib MK Palm G Dynamics of neuronal firing correlation: modulation of "effective connectivity" J Neurophysiol 1989 61 5 900 917 2723733 10.1152/jn.1989.61.5.900

[B5] Stephan KE Riera JJ Deco G Horwitz B The Brain Connectivity Workshops: moving the frontiers of computational systems neuroscience Neuroimage 2008 42 1 1 9 10.1016/j.neuroimage.2008.04.167 18511300 PMC2574909

[B6] Reijneveld JC Ponten SC Berendse HW Stam CJ The application of graph theoretical analysis to complex networks in the brain Clin Neurophysiol 2007 118 11 2317 2331 10.1016/j.clinph.2007.08.010 17900977

[B7] Bullmore E Sporns O Complex brain networks: graph theoretical analysis of structural and functional systems Nat Rev Neurosci 2009 10 3 186 198 10.1038/nrn2575 19190637

[B8] Sporns O Honey CJ Small worlds inside big brains Proc Natl Acad Sci USA 2006 103 51 19219 19220 10.1073/pnas.0609523103 17159140 PMC1748207

[B9] Nunez PL Nunez PL Neuromodulation of neocortical dynamics Neocortical dynamcs and human EEG rhythms 1995 Oxford: Oxford University Press

[B10] Watts DJ Strogatz SH Collective dynamics of 'small-world' networks Nature 1998 393 6684 440 442 10.1038/30918 9623998

[B11] He Y Chen ZJ Evans AC Small-world anatomical networks in the human brain revealed by cortical thickness from MRI Cereb Cortex 2007 17 10 2407 2419 10.1093/cercor/bhl149 17204824

[B12] Stam CJ Functional connectivity patterns of human magnetoencephalographic recordings: a 'small-world' network? Neurosci Lett 2004 355 1-2 25 28 10.1016/j.neulet.2003.10.063 14729226

[B13] Salvador R Suckling J Schwarzbauer C Bullmore E Undirected graphs of frequency-dependent functional connectivity in whole brain networks Philos Trans R Soc Lond B Biol Sci 2005 360 1457 937 946 10.1098/rstb.2005.1645 16087438 PMC1854928

[B14] Bosma I Douw L Bartolomei F Heimans JJ van Dijk BW Postma TJ Stam CJ Reijneveld JC Klein M Synchronized brain activity and neurocognitive function in patients with low-grade glioma: a magnetoencephalography study Neuro Oncol 2008 10 5 734 744 10.1215/15228517-2008-034 18650489 PMC2666250

[B15] Bartolomei F Bosma I Klein M Baayen JC Reijneveld JC Postma TJ Heimans JJ van Dijk BW de Munck JC de Jongh A How do brain tumors alter functional connectivity? A magnetoencephalography study Ann Neurol 2006 59 1 128 138 10.1002/ana.20710 16278872

[B16] Bartolomei F Bosma I Klein M Baayen JC Reijneveld JC Postma TJ Heimans JJ van Dijk BW de Munck JC de Jongh A Disturbed functional connectivity in brain tumour patients: evaluation by graph analysis of synchronization matrices Clin Neurophysiol 2006 117 9 2039 2049 10.1016/j.clinph.2006.05.018 16859985

[B17] Bosma I Reijneveld JC Klein M Douw L van Dijk BW Heimans JJ Stam CJ Disturbed functional brain networks and neurocognitive function in low-grade glioma patients: a graph theoretical analysis of resting-state MEG Nonlinear Biomed Phys 2009 3 1 9 10.1186/1753-4631-3-9 19698149 PMC2745411

[B18] Douw L de Groot M van Dellen E Heimans JJ Ronner HE Stam CJ Reijneveld JC 'Functional connectivity' is a sensitive predictor of epilepsy diagnosis after the first seizure PLoS One 2010 5 5 e10839 10.1371/journal.pone.0010839 20520774 PMC2877105

[B19] Horstmann MT Bialonski S Noennig N Mai H Prusseit J Wellmer J Hinrichs H Lehnertz K State dependent properties of epileptic brain networks: Comparative graph-theoretical analyses of simultaneously recorded EEG and MEG Clin Neurophysiol 2010 121 2 172 185 10.1016/j.clinph.2009.10.013 20045375

[B20] Douw L Baayen H Bosma I Klein M Vandertop P Heimans J Stam K de Munck J Reijneveld J Treatment-related changes in functional connectivity in brain tumor patients: A magnetoencephalography study Exp Neurol 2008 212 285 290 10.1016/j.expneurol.2008.03.013 18534578

[B21] van Dellen E Douw L Baayen JC Heimans JJ Ponten SC Vandertop WP Velis DN Stam CJ Reijneveld JC Long-term effects of temporal lobe epilepsy on local neural networks: a graph theoretical analysis of corticography recordings PLoS One 2009 4 11 e8081 10.1371/journal.pone.0008081 19956634 PMC2778557

[B22] Liao W Zhang Z Pan Z Mantini D Ding J Duan X Luo C Lu G Chen H Altered functional connectivity and small-world in mesial temporal lobe epilepsy PLoS One 2009 5 1 e8525 10.1371/journal.pone.0008525 PMC2799523 20072616

[B23] Gong G Rosa-Neto P Carbonell F Chen ZJ He Y Evans AC Age- and gender-related differences in the cortical anatomical network J Neurosci 2009 29 50 15684 15693 10.1523/JNEUROSCI.2308-09.2009 20016083 PMC2831804

[B24] Gootjes L Bouma A Van Strien JW Scheltens P Stam CJ Attention modulates hemispheric differences in functional connectivity: evidence from MEG recordings Neuroimage 2006 30 1 245 253 10.1016/j.neuroimage.2005.09.015 16253520

[B25] Ramasco JJ Goncalves B Transport on weighted networks: When the correlations are independent of the degree Phys Rev E Stat Nonlin Soft Matter Phys 2007 76 6 Pt 2 066106 18233897 10.1103/PhysRevE.76.066106

[B26] Morgan RJ Soltesz I Nonrandom connectivity of the epileptic dentate gyrus predicts a major role for neuronal hubs in seizures Proc Natl Acad Sci USA 2008 105 16 6179 6184 10.1073/pnas.0801372105 18375756 PMC2299224

[B27] Bonifazi P Goldin M Picardo MA Jorquera I Cattani A Bianconi G Represa A Ben-Ari Y Cossart R GABAergic hub neurons orchestrate synchrony in developing hippocampal networks Science 2009 326 5958 1419 1424 10.1126/science.1175509 19965761

[B28] Kramer MA Kolaczyk ED Kirsch HE Emergent network topology at seizure onset in humans Epilepsy Res 2008 79 2-3 173 186 10.1016/j.eplepsyres.2008.02.002 18359200

[B29] Ponten SC Bartolomei F Stam CJ Small-world networks and epilepsy: Graph theoretical analysis of intracerebrally recorded mesial temporal lobe seizures Clin Neurophysiol 2007 118 4 918 927 10.1016/j.clinph.2006.12.002 17314065

[B30] Schindler K Leung H Elger CE Lehnertz K Assessing seizure dynamics by analysing the correlation structure of multichannel intracranial EEG Brain 2007 130 Pt 1 65 77 17082199 10.1093/brain/awl304

[B31] Mizuki Y Tanaka M Isozaki H Nishijima H Inanaga K Periodic appearance of theta rhythm in the frontal midline area during performance of a mental task Electroencephalogr Clin Neurophysiol 1980 49 3-4 345 351 10.1016/0013-4694(80)90229-1 6158411

[B32] Kahana MJ The cognitive correlates of human brain oscillations J Neurosci 2006 26 6 1669 1672 10.1523/JNEUROSCI.3737-05c.2006 16467513 PMC6793637

[B33] Klimesch W EEG alpha and theta oscillations reflect cognitive and memory performance: a review and analysis Brain Res Brain Res Rev 1999 29 2-3 169 195 10.1016/S0165-0173(98)00056-3 10209231

[B34] Stam CJ van Cappellen van Walsum AM Micheloyannis S Variability of EEG synchronization during a working memory task in healthy subjects Int J Psychophysiol 2002 46 1 53 66 10.1016/S0167-8760(02)00041-7 12374646

[B35] Klausberger T Magill PJ Marton LF Roberts JD Cobden PM Buzsaki G Somogyi P Brain-state- and cell-type-specific firing of hippocampal interneurons in vivo Nature 2003 421 6925 844 848 10.1038/nature01374 12594513

[B36] Klausberger T Somogyi P Neuronal diversity and temporal dynamics: the unity of hippocampal circuit operations Science 2008 321 5885 53 57 10.1126/science.1149381 18599766 PMC4487503

[B37] Mackenzie L Medvedev A Hiscock JJ Pope KJ Willoughby JO Picrotoxin-induced generalised convulsive seizure in rat: changes in regional distribution and frequency of the power of electroencephalogram rhythms Clin Neurophysiol 2002 113 4 586 596 10.1016/S1388-2457(02)00040-8 11956004

[B38] Marcelin B Chauviere L Becker A Migliore M Esclapez M Bernard C h channel-dependent deficit of theta oscillation resonance and phase shift in temporal lobe epilepsy Neurobiol Dis 2009 33 3 436 447 10.1016/j.nbd.2008.11.019 19135151

[B39] Clemens B Pathological theta oscillations in idiopathic generalised epilepsy Clin Neurophysiol 2004 115 6 1436 1441 10.1016/j.clinph.2004.01.018 15134712

[B40] Mirsky AF Grady CF Myslobodsky MS, Mirsky AF Toward the development of alternative treatments in absence epilepsy Elements of petit mal epilepsy 1988 New York: Peter Lang 285 310

[B41] Clemens B Szigeti G Barta Z EEG frequency profiles of idiopathic generalised epilepsy syndromes Epilepsy Res 2000 42 2-3 105 115 10.1016/S0920-1211(00)00167-4 11074183

[B42] Clemens B Abnormal quantitative EEG scores identify patients with complicated idiopathic generalised epilepsy Seizure 2004 13 6 366 374 10.1016/j.seizure.2003.09.012 15276139

[B43] Veauthier J Haettig H Meencke HJ Impact of levetiracetam add-on therapy on different EEG occipital frequencies in epileptic patients Seizure 2009 18 6 392 395 10.1016/j.seizure.2009.02.001 19269864

[B44] Smit DJ Stam CJ Posthuma D Boomsma DI de Geus EJ Heritability of "small-world" networks in the brain: A graph theoretical analysis of resting-state EEG functional connectivity Hum Brain Mapp 2008 29 12 1368 1378 10.1002/hbm.20468 18064590 PMC6870849

[B45] Posthuma D de Geus EJ Mulder EJ Smit DJ Boomsma DI Stam CJ Genetic components of functional connectivity in the brain: the heritability of synchronization likelihood Hum Brain Mapp 2005 26 3 191 198 10.1002/hbm.20156 15929086 PMC6871713

[B46] Karnofsky DA Abelmann WH Craver LF The use of nitrogen mustards in the palliative treatment of carcinoma Cancer 1948 1 634 656 10.1002/1097-0142(194811)1:4<634::AID-CNCR2820010410>3.0.CO;2-L

[B47] Wade DT Collin C The Barthel ADL Index: a standard measure of physical disability? Int Disabil Stud 1988 10 2 64 67 3042746 10.3109/09638288809164105

[B48] Vrba J Anderson G Betts K Yoshimoto T, Kotani M, Kuriki S 151-Channel whole-cortex MEG system for seated or supine positions Recent Advances in Biomagnetism 1999 Sendai, Japan: Tohoku University Press 93 96

[B49] Stam CJ Jones BF Manshanden I van Cappellen van Walsum AM Montez T Verbunt JP de Munck JC van Dijk BW Berendse HW Scheltens P Magnetoencephalographic evaluation of resting-state functional connectivity in Alzheimer's disease Neuroimage 2006 32 3 1335 1344 10.1016/j.neuroimage.2006.05.033 16815039

[B50] Stam CJ Nolte G Daffertshofer A Phase lag index: assessment of functional connectivity from multi channel EEG and MEG with diminished bias from common sources Hum Brain Mapp 2007 28 11 1178 1193 10.1002/hbm.20346 17266107 PMC6871367

[B51] Rubinov M Sporns O Complex network measures of brain connectivity: Uses and interpretations Neuroimage 2010 52 3 1059 1069 10.1016/j.neuroimage.2009.10.003 19819337

[B52] Stam CJ de Haan W Daffertshofer A Jones BF Manshanden I van Cappellen van Walsum AM Montez T Verbunt JP de Munck JC van Dijk BW Graph theoretical analysis of magnetoencephalographic functional connectivity in Alzheimer's disease Brain 2009 132 Pt 1 213 224 18952674 10.1093/brain/awn262

[B53] Humphries MD Gurney K Network 'small-world-ness': a quantitative method for determining canonical network equivalence PLoS ONE 2008 3 4 e0002051 10.1371/journal.pone.0002051 18446219 PMC2323569

[B54] Nolte G Bai O Wheaton L Mari Z Vorbach S Hallett M Identifying true brain interaction from EEG data using the imaginary part of coherency Clin Neurophysiol 2004 115 10 2292 2307 15351371 10.1016/j.clinph.2004.04.029

